# Predictors of clozapine concentration and psychiatric symptoms in patients with schizophrenia

**DOI:** 10.1371/journal.pone.0319037

**Published:** 2025-03-06

**Authors:** Sang-In Park, Seoyoung Kim, Kwanwoo Park, Uijeong Yu, Yunjeong Jang, Bo-Hyung Kim, Ji Hyun Lee, Euitae Kim

**Affiliations:** 1 Department of Pharmacology, College of Medicine, Kangwon National University, Chuncheon, Republic of Korea; 2 Biomedical Research Institute, Kangwon National University Hospital, Chuncheon, Republic of Korea; 3 Department of Neuropsychiatry, Seoul National University Bundang Hospital, Seongnam, Republic of Korea; 4 Department of Clinical Pharmacology and Therapeutics, College of Medicine, Kyung Hee University, Seoul, Republic of Korea; 5 Department of Biomedical and Pharmaceutical Sciences, Graduate School, Kyung Hee University, Seoul, Republic of Korea; 6 RexSoft Corp., Seoul, Republic of Korea; 7 East-West Medical Research Institute, Kyung Hee University, Seoul, Republic of Korea; 8 Department of Clinical Pharmacology and Therapeutics, Kyung Hee University Hospital, Seoul, Republic of Korea; 9 Department of Biomedical Science and Technology, Kyung Hee University, Seoul, Republic of Korea; 10 Department of Biomedical and Pharmaceutical Sciences, Kyung Hee University, Seoul, Republic of Korea; 11 Department of Precision Medicine, Graduate School, Kyung Hee University, Seoul, Republic of Korea; 12 Department of Brain and Cognitive Sciences, Seoul National University College of Natural Sciences, Seoul, Republic of Korea; 13 Department of Psychiatry, Seoul National University College of Medicine, Seoul, Republic of Korea; Case Western Reserve University, UNITED STATES OF AMERICA

## Abstract

Clozapine has superior efficacy to other antipsychotics, especially in patients with treatment-resistant schizophrenia. However, its pharmacokinetics and pharmacodynamics vary largely among patients. We aimed to evaluate the clinical and genetic factors associated with the pharmacokinetics and pharmacodynamics of clozapine in patients with schizophrenia. Blood samples for clozapine pharmacokinetic assessment were collected from patients with schizophrenia at weeks 2 (visit 2), 8 (visit 3), and 18 (visit 4) from the initiation of clozapine treatment. The Positive and Negative Syndrome Scale (PANSS) score was assessed at baseline (visit 1) and visits 3 and 4. Linear mixed models were used to identify the clinical and genetic variables associated with the clozapine concentration and total PANSS score. A total of 45 patients were included in the pharmacogenomic analysis. Owing to the small sample size, we categorized concomitant medications into four groups. However, individual drugs may have different effects on clozapine concentration. Clozapine concentration was significantly associated with smoking status and cumulative clozapine dose. Clozapine concentration was significantly associated with five single nucleotide polymorphisms (SNPs) in three genes (rs28371726 and rs202102799 in *CYP2D6*, rs4148323 and rs34946978 in *UGT1A1*, and rs2011404 in *UGT1A4*). Furthermore, follow-up time, body mass index, and total bilirubin levels were significantly associated with the total PANSS scores. The PANSS score was significantly associated with four SNPs in two genes (rs7787082 and rs10248420 in *ABCB1* and rs2133251840 and rs762502 in *DRD4*). This study suggests potential clinical and genetic predictors of clozapine concentration and psychiatric symptoms in patients with schizophrenia treated with clozapine. With further investigations in diverse populations, our findings may provide important information on variables to be considered in individualized clozapine treatment.

## Introduction

Schizophrenia is a complex and debilitating mental disorder involving impairment of cognitive abilities and social functions, with symptoms typically presenting in late adolescence or early adulthood [1,2]. Antipsychotic medications in conjunction with nonpharmacological treatments such as psychotherapy are the mainstay of schizophrenia treatment [3]. However, approximately 20–30% of patients have treatment-resistant schizophrenia wherein they do not satisfactorily respond to two or more trials of antipsychotic medications at the adequate dose and duration [4].

Clozapine is the only approved medication for the management of treatment-resistant schizophrenia, with approximately 40% patients responding adequately [5,6]. It has also been found to be clinically beneficial in patients with a high risk of suicide, aggressive behavior, and concurrent substance use disorders [7]. However, despite its superior effectiveness to other antipsychotics, especially for patients with treatment-resistant schizophrenia, there is still reluctance to prescribe it due to several reasons, such as concerns about blood level monitoring or its potential adverse effects [8,9]. Moreover, considering the large inter-individual variability in the pharmacokinetics and pharmacodynamics of clozapine, the individual factors affecting clozapine concentration, and the treatment response need to be considered.

Clozapine undergoes extensive hepatic metabolism via two main pathways: demethylation to N-desmethylclozapine (NDMC) and oxidation to clozapine N-oxide [10]. Several cytochrome P450 (CYP) enzymes, including CYP1A2, CYP3A4, CYP2D6, and CYP2C19, are involved in clozapine metabolism, with CYP1A2 having a major role *in vivo* [10]. In addition, uridine diphosphate-glucuronosyltransferase (UGT) enzymes are also involved in clozapine metabolism by catalyzing the formation of clozapine-glucuronide metabolites [11].

The diversity in the response to clozapine has been attributed to a wide range of neurotransmitter receptors with which clozapine interacts, including dopamine, serotonin, adrenergic, histaminergic, and muscarinic receptors [12,13]. Moreover, some of the actions of NDMC, an active metabolite of clozapine, are opposite to those of clozapine. For example, NDMC acts as a partial agonist at the M1 muscarinic receptor, in contrast to the antagonistic effect of clozapine on the same receptor [14]. In these contexts, the clozapine**/**NDMC ratio is a useful indicator not only for assessing medication adherence and making dose adjustments but also for predicting response to clozapine and identifying its adverse effects [14].

Clinical factors associated with clozapine concentration have been investigated, but the results have been inconsistent [15,16]. For example, some studies have reported the influence of age on clozapine plasma concentrations [17,18], while others have not confirmed these findings [19,20]. The identified clinical markers associated with the response to clozapine treatment have also varied. For instance, a study reported that an increase in serum triglyceride levels was a significant predictor for improvement in Positive and Negative Syndrome Scale (PANSS) Positive and Negative scores [21]. Meanwhile, another study suggested that a combination of markers, including monocyte/lymphocyte ratio, mean platelet volume, platelet/lymphocyte ratio, and unconjugated bilirubin, could be used to predict acute stage of schizophrenia [22].

In addition to clinical variables, the evidence on genetic factors associated with clozapine concentration or treatment response to clozapine has also been inconsistent [23,24]. One study failed to find a significant relationship between Ser9Gly polymorphism in the dopamine D3 receptor gene (*DRD3*) in response to clozapine treatment [25], whereas another study reported that the Gly-9 allele of the *DRD3* Ser9Gly polymorphism was significantly associated with treatment response to clozapine [26]. Considering these findings, investigating the clinical and genetic markers associated with individual clozapine pharmacokinetics and pharmacodynamics may help create an optimal clozapine dosing plan. Therefore, this study aimed to evaluate the clinical and genetic factors associated with clozapine concentration and severity of psychiatric symptoms as evaluated using the PANSS.

## Materials and methods

### Study design and patients

This prospective observational study was conducted for patients recruited from November 27, 2018 to January 27, 2021. Patients aged ≥ 19 years who were diagnosed with schizophrenia and other psychotic disorders according to the Diagnostic and Statistical Manual of Mental Disorders, fifth edition, and were expected to use clozapine for treatment were enrolled.

Patient characteristics, including body weight, body mass index (BMI), and concomitant medications, were assessed at baseline (visit 1) and at weeks 2 (visit 2), 8 (visit 3), and 18 (visit 4) from the initiation of clozapine dosing. The clozapine dose-titration schedule was individualized by clinicians based on the patient’s clinical status, with close monitoring of adverse effects. Symptom severity and treatment response were assessed using the total score of the 30-item PANSS, based on clinical interviews performed at baseline (visit 1) and at weeks 8 (visit 3) and 18 (visit 4) from the initiation of clozapine dosing. Clinical laboratory tests to assess the adverse effects of clozapine treatment were conducted at baseline (visit 1) and at weeks 2 (visit 2) and 8 (visit 3) from the initiation of clozapine treatment.

The study protocol was approved by the Institutional Review Board of the Seoul National University Bundang Hospital (IRB Number: B-1805/471-307). The study was conducted in compliance with the ethical principles of the Declaration of Helsinki and Korean Good Clinical Practice and registered at the Clinical Research Information Service (https://cris.nih.go.kr) as KCT0003052. Written informed consent was obtained from all patients or their legal guardian.

### Plasma clozapine and NDMC concentrations

Blood samples for clozapine pharmacokinetic assessment were collected with information on the cumulative dose of clozapine at weeks 2 (visit 2), 8 (visit 3), and 18 (visit 4) after the initiation of clozapine treatment. Plasma clozapine and NDMC concentrations were determined using the validated liquid chromatography coupled with tandem mass spectrometry (LC-MS/MS) method using D4-clozapine (Merck, Rahway, NJ, USA) as an internal standard, as reported previously [27]. Briefly, the blood samples were centrifuged at 2,000 × g for 10 min at 4°C. The supernatants were injected into an AB Sciex 6500 Triple Quad LC-MS/MS System (AB Sciex, Framingham, MA, USA). The analytes were separated using an ACQUITY UPLC BEH C18 column (2.1 × 50 mm, 1.7 µm; Waters Corporation, Milford, MA, USA) at 25°C. Quantification was performed in the multiple-reaction monitoring mode to monitor the transitions (*m/z*) of 327.15–270.00 for clozapine and 313.15–192.00 for NDMC. The lower limit of quantification for both clozapine and NDMC was 5 ng/mL. The calibration curves were linear over the range of 5–1,000 ng/mL for clozapine and 5–500 ng/mL for NDMC.

### Pharmacogenomic analysis

A blood sample (3 mL) for exploratory genetic analysis was collected from each patient and stored at –20°C until analysis. Genomic deoxyribonucleic acid was extracted from blood samples. Single nucleotide polymorphisms (SNPs) potentially related to clozapine pharmacokinetics or treatment response were included in the analysis. Specifically, previously reported SNPs in the *CYP* and *UGT* genes were selected for pharmacokinetic analysis, while SNPs in the histaminergic receptor (*HRH1*), dopaminergic receptors (*DRD2*, *DRD3*, *DRD4*), adenosine triphosphate-binding cassette subfamily B member 1 (*ABCB1*), and catechol-O-methyltransferase (*COMT*) genes were selected for pharmacodynamic analysis using targeted next-generation sequencing. Hybridization capture-based next-generation sequencing was performed using the Illumina NextSeq 550 platform (Illumina, San Diego, CA, USA) by Celemics Inc. (Seoul, Republic of Korea). Sequencing data were analyzed using a genome analysis tool kit.

### Statistical analysis

Baseline patient characteristics were presented using descriptive statistics. For continuous variables, the Student’s *t*-test or Mann–Whitney U test was used to test the differences between groups classified by sex or smoking status. For categorical variables, the chi-square test or Fisher’s exact test was used. A linear mixed model was developed to identify variables significantly associated with the pharmacokinetics (clozapine concentration as a dependent variable) or pharmacodynamics (the total PANSS score as a dependent variable) of clozapine. Independent variables included demographic data, follow-up time, metabolic ratio (clozapine/NDMC ratio; included as an independent variable for the total PANSS score), clinical laboratory test results, and medication factors (including cumulative doses of concomitant antipsychotics calculated as olanzapine equivalents collected at each visit). Plasma concentrations of clozapine and doses of clozapine or concomitant antipsychotics were log-transformed for analysis. Genetic variations of each SNP (wild-type, heterozygous groups, and homozygous mutant group) were coded as 0, 1, and 2 for linear mixed model analyses. Each variable was evaluated using a univariable model. Subsequently, multivariable analysis was performed including the significant variables identified in the univariable analysis with consideration of their clinical significance. A *p*-value of < 0.05 was considered statistically significant. All statistical analyses were performed using SPSS 26.0 (SPSS Inc., Chicago, IL, USA) and R software (version 3.6.3; R Foundation for Statistical Computing, Vienna, Austria).

## Results

### Patient characteristics

**Fig 1:**
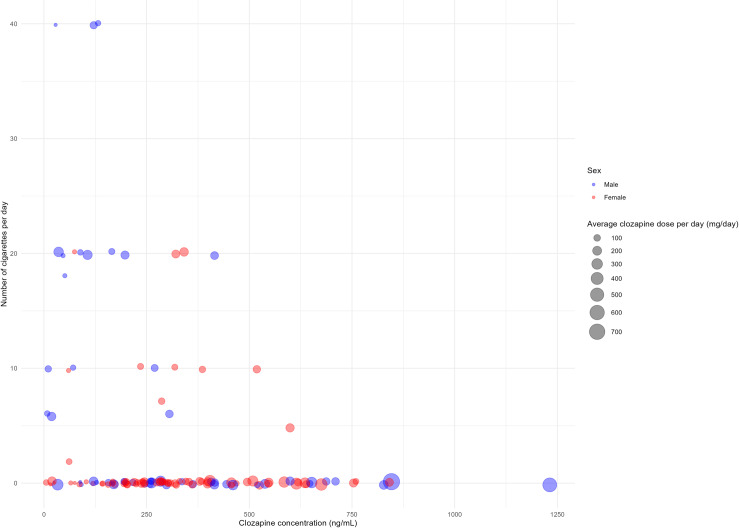
Scatterplot illustrating the relationship between the number of cigarettes per day, clozapine concentration, clozapine dose, and sex.

A total of 45 patients who met the eligibility criteria were enrolled in this study. The mean duration of illness from onset was 2,870 days, and the mean ( ± standard deviation) baseline total PANSS score for the 42 patients who could be assessed was 82.24 ± 16.13. The mean height and weight significantly differed between male and female patients and between smokers and non-smokers (*p* < 0.05, t-test; Table 1). The total PANSS and PANSS Positive and Negative subscale scores at baseline were significantly higher in males than in females (*p* < 0.05, t-test; Table 1). Of the 45 patients, two dropped out before visit 3 and another two before visit 4. Therefore, a total of 41 patients completed the study. The average daily clozapine doses were 76.4 ± 36.1 mg/day, 153.6 ± 120.9 mg/day, and 180.3 ± 95.3 mg/day at weeks 2 (visit 2), 8 (visit 3), and 18 (visit 4) from the initiation of clozapine dosing, respectively. During the study period, the minimum daily dose observed was 19.2 mg/day, while the maximum daily dose reached 790.9 mg/day (S1 Table and Fig 1).

**Table 1:** Baseline patient characteristics.

Variable	Total (*n* = 45)	Sex			Smoking status		
		Male (*n* = 18)	Female (*n* = 27)	*p*-value	Smoker (*n* = 11)	Non-smoker (*n* = 34)	*p*-value
Age (years)	35.40 ± 10.43	32.11 ± 8.87	37.59 ± 10.97	0.054^a^	34.82 ± 9.68	35.59 ± 10.80	0.864^a^
Height (cm)	165.82 ± 8.51	174.29 ± 5.58	160.18 ± 4.41	**<0.001**	171.03 ± 9.06	164.14 ± 7.73	**0.018**
Weight (kg)	69.42 ± 17.71	80.15 ± 17.16	62.26 ± 14.34	**<0.001**	81.47 ± 20.34	65.52 ± 15.13	**0.008**
BMI (kg/m^2^)	25.07 ± 5.26	26.36 ± 5.40	24.20 ± 5.09	0.181	27.63 ± 5.66	24.24 ± 4.93	0.063
Female (%)	27 (60.00%)				4 (36.36%)	23 (67.65%)	0.086^b^
Smoking status, n (%)				0.086^b^			
No	34 (75.56%)	11 (61.11%)	23 (85.19%)				
Yes	11 (24.44%)	7 (38.89%)	4 (14.81%)				
Caffeine intake status, n (%)				1.000			**0.033** ^b^
No	17 (37.78%)	7 (38.89%)	10 (37.04%)		1 (9.09%)	16 (47.06%)	
Yes	28 (62.22%)	11 (61.11%)	17 (62.96%)		10 (90.91%)	18 (52.94%)	
Total PANSS^c^	82.24 ± 16.13	89.06 ± 20.19	77.60 ± 10.82	**0.043**	86.90 ± 19.82	80.78 ± 14.86	0.506^a^
Positive PANSS	19.83 ± 5.51	22.29 ± 6.78	18.16 ± 3.75	**0.032**	21.30 ± 7.04	19.38 ± 4.99	0.341
Negative PANSS	19.57 ± 3.91	21.24 ± 4.68	18.44 ± 2.86	**0.038**	20.20 ± 3.82	19.38 ± 3.97	0.624^a^
General PANSS	42.83 ± 8.82	45.53 ± 10.87	41.00 ± 6.75	0.139	45.40 ± 9.99	42.03 ± 8.44	0.298

^a^Mann–Whitney U test.

^b^Fisher’s exact test.

^c^The data of 42 patients who could be assessed at baseline are included.

Data are reported as the mean ±  SD for continuous variables and n (%) for categorical variables. *p*-values are computed by the t-test or Mann–Whitney U test for continuous variables and the chi-square test or Fisher’s exact test for categorical variables, as appropriate. Bold values denote statistical significance (*p* < 0.05).

BMI, body mass index; PANSS, Positive and Negative Syndrome Scale.

### Pharmacogenomic assessment associated with clozapine metabolic ratio

Of the 45 enrolled patients, 17 were normal metabolizers (NMs) of CYP1A2 (*1A/ * 1A, * 1C * 1F/ * 1C * 1F, * 1C/ * 1F, or * 1A/ * 1C * 1F), while 28 were ultrarapid metabolizers (UMs) (*1F/ * 1F, * 1F/ * 1C * 1F, or * 1A/ * 1F). Regarding CYP2C19, 20 patients were NMs (*1/ * 1), 19 were intermediate metabolizers (*1/ * 2), and six were poor metabolizers (PMs) (*2/ * 2). CYP1A2 phenotypes were determined by a combination of two SNPs, namely rs2069514 (c.-3860G > A) and rs762551 (c.-9-154C > A). In addition, CYP2C19 phenotypes were determined by three linked SNPs, namely rs12769205 (c.332-23A > G), rs4244285 (c.681G > A), and rs3758580 (c.990C > T) (S2 Table).

CYP1A2 phenotypes were significantly associated with the metabolic ratio (clozapine/NDMC ratio) (*p* = 0.006, Mann–Whitney U test) at visit 4, with higher metabolic ratios in NMs (*n* = 14) than that in UMs (*n* = 26) (S1 Fig). Meanwhile, CYP2C19 phenotypes were significantly associated with the metabolic ratio (*p* = 0.033, Kruskal–Wallis test) at visit 2, with a higher metabolic ratio in PMs (*n* = 6) than that in NMs and IMs (*n* = 38) (*p* = 0.013, Mann–Whitney U test) (S2 Fig).

### Clinical variables associated with clozapine concentration

In the univariable analysis, follow-up time and demographic factors, including age and smoking status, were significantly associated with clozapine concentration. Among medication factors, the cumulative dose of clozapine, number of concomitant psychotropic agents categorized as “others,” and cumulative doses of concomitant antipsychotics calculated as olanzapine equivalents were found to be significant. However, when we individually analyzed each concomitant medication, the number of patients taking each medication was too small to yield significant results. The list of concomitant psychotropic agents and the number of patients who received them are shown in S3 Table. Among the clinical laboratory results, triglyceride levels were statistically significant in the univariable model. Based on these results, the smoking status and cumulative dose of clozapine were included as significant variables in the final multivariable model for the predictors of clozapine concentration (Table 2). Further, compared with non-smokers, smokers exhibited lower clozapine and NDMC concentrations at visits 2 and 3. Similar results were found at visit 4, although no statistical significance was observed in clozapine concentration (S1 Table).

**Table 2:** Factors associated with clozapine concentration in the linear mixed model.

Variable	Univariable model			Candidate multivariable model			Final multivariable model		
	Beta	(95% CI)	*p*-value	Beta	(95% CI)	*p*-value	Beta	(95% CI)	*p*-value
**Visit**									
Follow-up time (days)	**0.004**	**(0.001, 0.007)**	**0.013**	–0.012	(–0.024, 0.000)	0.069			
**Demographics**									
Age (years)	**–0.021**	**(–0.041, –0.001)**	**0.043**	–0.009	(–0.023, 0.005)	0.228			
Sex (female)	0.231	(–0.205, 0.667)	0.301						
Height (cm)	–0.019	(–0.045, 0.006)	0.135						
Weight (kg)	–0.011	(–0.023, 0.001)	0.072						
BMI (kg/m^2^)	–0.027	(–0.068, 0.014)	0.203						
Smoking status (Yes)	**–0.874**	**(–1.339, –0.414)**	**<0.001**	**–0.829**	**(–1.191, –0.455)**	**<0.001**	**–0.853**	**(–1.287, –0.428)**	**<0.001**
Concomitant medication (Yes)	0.085	(–0.769, 0.939)	0.845						
**Clozapine**									
Clozapine dose (cumulative, mg)	**0.238**	**(0.134, 0.345)**	**<0.001**	**0.464**	**(0.250, 0.681)**	**<0.001**	**0.238**	**(0.139, 0.341)**	**<0.001**
**Type of concomitant psychotropic agents** ^a^									
Antidepressant	0.244	(–0.119, 0.607)	0.191						
Anxiolytic	0.012	(–0.284, 0.311)	0.935						
Mood stabilizer	–0.415	(–0.845, 0.014)	0.061						
Others	**0.230**	**(0.094, 0.366)**	**0.001**	0.074	(–0.037, 0.179)	0.193			
**Dose equivalence of concomitant antipsychotic drugs** ^b^									
Doses of antipsychotic drugs	–0.024	(–0.175, 0.127)	0.752						
Cumulative dose of antipsychotic drugs	**0.144**	**(–0.002, 0.292)**	**0.048**	–0.006	(–0.129, 0.115)	0.922			
**Laboratory test results**									
Triglyceride	**–0.003**	**(–0.005, –0.001)**	**0.014**	–0.001	(–0.003, 0.001)	0.210			
ALT	0.002	(–0.002, 0.006)	0.395						
AST	0.003	(–0.005, 0.010)	0.461						
Albumin	0.070	(–0.674, 0.819)	0.852						
Total bilirubin	0.233	(–0.436, 0.900)	0.491						
Creatinine	–1.112	(–2.389, 0.188)	0.089						
**CYP1A2 phenotype**									
NM	0	(reference)							
UM	0.054	(–0.394, 0.502)	0.814						

^a^The number of concomitant medications within each category was used.

^b^Olanzapine equivalent dose calculated in previous publications [28,29].

Bold values denote statistical significance (*p* < 0.05).

ALT, alanine transaminase; AST, aspartate transaminase; BMI, body mass index; CI, confidence interval; CYP, cytochrome P450; NM, normal metabolizer; UM, ultrarapid metabolizer.

### Genetic factors associated with clozapine concentration

Of the 81 pharmacokinetics-related variations (S4 Table), nine SNPs (seven in *CYP2D6*, one in *UGT1A1*, and one in *UGT1A4*) were significantly associated with clozapine concentration. In the model adjusted for clinical predictors of clozapine concentration, including smoking status and cumulative dose of clozapine, five SNPs (rs28371726 and rs202102799 in *CYP2D6*; rs4148323 and rs34946978 in *UGT1A1*; and rs2011404 in *UGT1A4*) showed significant associations with clozapine concentration. The rs number for each SNP associated with clozapine concentration is shown in Table 3.

**Table 3:** Association of SNPs with the clozapine concentration in the linear mixed model.

Gene	RSID	Base change^b^	Crude model			Adjusted model^c^		
			Beta	(95% CI)	*p*-value	Beta	(95% CI)	*p*-value
CYP2D6	rs1135840^a^	c.1457C > G	**–0.356**	**(–0.646, –0.066)**	**0.018**	–0.202	(–0.458, 0.055)	0.130
	rs61745683	c.1108G > A	**–0.611**	**(–1.198, –0.018)**	**0.044**	–0.413	(–0.917, 0.095)	0.115
	rs28371726	c.1083T > C	**–1.343**	**(–1.906, –0.779)**	**<0.001**	**–1.066**	**(–1.555, –0.576)**	**<0.001**
	rs202102799	c.1064A > G	**–1.558**	**(–2.270, –0.846)**	**<0.001**	**–1.122**	**(–1.776, –0.466)**	**0.001**
	rs1058164^a^	c.408C > G	**–0.336**	**(–0.630, –0.040)**	**0.028**	–0.195	(–0.452, 0.063)	0.146
	rs28371702^a^	n. * 4598C > A	**–0.356**	**(–0.646, –0.066)**	**0.018**	–0.202	(–0.458, 0.055)	0.130
	rs28371699^a^	c.180 + 130T > G	**–0.356**	**(–0.646, –0.066)**	**0.018**	–0.202	(–0.458, 0.055)	0.130
UGT1A1	rs4148323	c.211G > A	–0.439	(–0.957, 0.080)	0.100	**–0.656**	**(–1.059, –0.254)**	**0.002**
	rs34946978	c.1082C > T	**–2.495**	**(–3.725, –1.266)**	**<0.001**	**–1.921**	**(–3.010, –0.827)**	**0.001**
UGT1A4	rs2011404	c.471T > C	**1.563**	**(0.197, 2.929)**	**0.027**	**1.159**	**(0.492, 2.706)**	**0.006**

^a^Linkage disequilibrium with r^2^ > 0.8. Bold values denote statistical significance (*p* < 0.05).

^b^Nucleotide location numbers are assigned according to *CYP2D6* (NM_000106.5), *UGT1A1* (NM_000463.2), and *UGT1A4* (NM_007120.2) mRNA sequences.

^c^Adjusted for smoking status and cumulative dose of clozapine.

CI, confidence interval; CYP, cytochrome P450; RSID, Reference Single nucleotide polymorphism-cluster IDentification; UGT, uridine diphosphate-glucuronosyltransferase.

### Variables associated with psychiatric symptoms

In the univariable analysis, follow-up time and demographic factors, including sex, height, and BMI, were significantly associated with the total PANSS score. Among the medication factors, the cumulative dose of clozapine and the number of concomitant antidepressants were found to be significant. In addition, equivalent doses of concomitant antipsychotics at each visit and their cumulative values until each visit were significantly associated with the PANSS score. Among the blood laboratory results, glucose, insulin, homeostatic model assessment of insulin resistance (HOMA-IR), high-density lipoprotein, total bilirubin, and leptin levels were significantly associated with the total PANSS score. Owing to differences in the data collection times of clozapine doses and clinical laboratory tests, they were included as independent variables in separate multivariable linear mixed models for predictors of the total PANSS score. Consequently, follow-up time, height, and BMI were significant in the multivariable model 1, while height and total bilirubin were significant in the multivariable model 2 (Table 4).

**Table 4:** Factors associated with the total PANSS score in the linear mixed model.

Variable	Univariable model			Multivariable model 1			Multivariable model 2		
	Beta	(95% CI)	*p*-value	Beta	(95% CI)	*p*-value	Beta	(95% CI)	*p*-value
**Visit**									
Follow-up time (days)	**–0.100**	**(–0.126, –0.075)**	**<0.001**	**–0.132**	**(–0.213, –0.050)**	**0.003**	0.080	(**–0.**153, 0.312)	0.533
**Demographics**									
Age (years)	–0.055	(–0.408, 0.296)	0.759						
Sex (female)	**–9.988**	**(–16.869, –3.132)**	**0.005**	1.165	(–7.970, 10.321)	0.814	3.103	(–9.085, 15.247)	0.646
Height (cm)	**0.711**	**(0.332, 1.089)**	**<0.001**	**0.773**	**(0.241, 1.308)**	**0.009**	**0.837**	**(0.205, 1.469)**	**0.019**
Weight (kg)	–0.066	(–0.287, 0.145)	0.533						
BMI (kg/m^2^)	**–0.821**	**(–1.547, –0.124)**	**0.020**	**–0.591**	**(–1.101, –0.081)**	**0.036**	**–0.**395	(–1.213, 0.420)	0.384
Smoking status (Yes)	1.546	(–6.621, 9.729)	0.712						
Caffeine intake status (Yes)	–3.536	(–9.182, 1.909)	0.181						
Concomitant medication (Yes)	–8.790	(–21.587, 4.050)	0.182						
**Clozapine PK**									
Clozapine dose (cumulative, mg)	**–4.176**	**(–6.473, –1.725)**	**<0.001**	3.655	(–1.136, 8.408)	0.160			
Metabolic ratio	–1.006	(–4.259, 2.096)	0.522						
**Type of concomitant psychotropic agents** ^a^									
Antidepressant	**–6.754**	**(–12.031, –1.428)**	**0.012**	–3.238	(–6.948, 0.626)	0.111	1.403	(–4.411, 7.224)	0.663
Anxiolytic	2.935	(–1.131, 6.963)	0.149						
Mood stabilizer	–3.175	(–9.849, 3.509)	0.348						
Others	–0.562	(–2.950, 1.867)	0.644						
**Dose equivalence of concomitant antipsychotic drugs** ^b^									
Doses of antipsychotic drugs (mg)	**3.315**	**(1.532, 4.990)**	**<0.001**	–0.819	(–2.820, 1.163)	0.440	0.814	(–2.396, 3.893)	0.635
Cumulative dose of antipsychotic drugs (mg)	**–2.574**	**(–3.355, –1.791)**	**<0.001**	–1.410	(–3.771, 0.902)	0.263	–2.669	(–5.833, 0.504)	0.131
Laboratory test results									
Glucose (mg/dL)	**–0.209**	**(–0.408, –0.002)**	**0.042**				0.048	(–0.316, 0.412)	0.812
Insulin (μIU/mL)	**–0.363**	**(–0.684, –0.044)**	**0.029**				–0.082	(–1.802, 1.663)	0.931
Glucagon (pg/mL)	–0.006	(–0.026, 0.015)	0.570						
HOMA-IR	–**1.262**	(–**2.337, –0.195)**	**0.023**				–0.259	(–6.794, 6.191)	0.942
Total cholesterol (mg/dL)	–0.046	(–0.142, 0.050)	0.354						
HDL (mg/dL)	**–0.266**	**(–0.525, –0.008)**	**0.047**				–0.143	(–0.392, 0.107)	0.306
LDL (mg/dL)	–0.019	(–0.140, 0.102)	0.754						
Triglyceride (mg/dL)	–0.015	(–0.062, 0.034)	0.545						
ALT (IU/L)	–0.008	(–0.097, 0.086)	0.859						
AST (IU/L)	0.104	(–0.122, 0.347)	0.333						
Albumin (g/dL)	–0.204	(–13.549, 13.530)	0.975						
Total bilirubin (mg/dL)	**17.575**	**(8.492, 26.695)**	**<0.001**				**9.854**	**(1.263, 18.579)**	**0.041**
Creatinine (mg/dL)	9.560	(–14.547, 33.656)	0.438						
Leptin (ng/mL)	**–0.319**	**(–0.557, –0.081)**	**0.010**				–0.105	(–0.383, 0.170)	0.493

^a^The number of concomitant medications within each category was used.

^b^Olanzapine equivalent dose calculated in previous publications [28,29].

Bold values denote statistical significance (*p* < 0.05).

ALT, alanine transaminase; AST, aspartate transaminase; BMI, body mass index; CI, confidence interval; HDL, high-density lipoprotein; HOMA-IR, homeostatic model assessment of insulin resistance; LDL, low-density lipoprotein; PK, pharmacokinetics.

### Genetic factors associated with the total PANSS score

Of the 75 SNPs screened for genetic analysis (S5 Table), one SNP (rs58224139) was excluded because all patients had the wild-type variant. Therefore, 74 variations in 15 genes, including *HRH1*, *DRD2*, *DRD3*, *DRD4*, *ABCB1*, and *COMT*, were investigated for their association with the PANSS score. Among these, five SNPs in three genes were significantly associated with the total PANSS score in the crude model: rs7787082 and rs10248420 in *ABCB1*, rs2133251840 and rs2133251864 in *DRD4*, and rs4818 in *COMT*. In the model adjusted for follow-up time and BMI, which were significant variables associated with the total PANSS score, four SNPs in two genes were significantly associated with the total PANSS score: rs7787082 and rs10248420 in *ABCB1* and rs2133251840 and rs762502 in *DRD4* (Table 5). Among these, only one SNP in *DRD4* (rs2133251840) resulted in different total PANSS scores at visits 3 and 4 according to its genotype (S6 Table).

**Table 5:** SNPs associated with the PANSS score in the linear mixed model.

Gene	RSID	Base change^b^	Crude model			Adjusted model^c^		
			Beta	(95% CI)	*p*-value	Beta	(95% CI)	*p*-value
*ABCB1*	rs7787082^a^	c.2685 + 3559C > T	**–5.491**	**(–10.568, –0.433)**	**0.036**	**–5.569**	**(–10.590, –0.563)**	**0.033**
	rs10248420^a^	c.2481 + 788T > C	**–5.491**	**(–10.568, –0.433)**	**0.036**	**–5.569**	**(–10.590, –0.563)**	**0.033**
*DRD4*	rs2133251840	c.710_711delGA	**11.545**	**(3.739, 19.371)**	**0.005**	**11.466**	**(3.770, 19.164)**	**0.005**
	rs2133251864	c.715_719delAGCGG	**–7.546**	**(–14.839, –0.260)**	**0.045**	**–**7.060	(**–**14.347, 0.241)	0.063
	rs762502	c.870C > T	–5.050	(–10.443, 0.354)	0.070	–**6.202**	(–**11.516, –0.900)**	**0.025**
*COMT*	rs4818	c.408C > G	**5.660**	**(0.201, 11.123)**	**0.045**	3.952	(–2.013, 9.859)	0.198

^a^Linkage disequilibrium with r^2^ > 0.8. Bold values denote statistical significance (*p* < 0.05).

^b^Nucleotide location numbers are assigned according to *ABCB1* (NM_000927.4), *DRD4* (NM_000797.3), and *COMT* (NM_000754.3) mRNA sequences.

^c^Adjusted for follow-up time and body mass index.

*ABCB1*, adenosine triphosphate-binding cassette subfamily B member 1; CI, confidence interval; *COMT*, catechol-O-methyltransferase; *DRD4*, dopamine D4 receptor gene, RSID, Reference Single nucleotide polymorphism-cluster IDentification.

## Discussion

This study identified the clinical and genetic variables significantly associated with clozapine concentration and psychiatric symptoms in patients with schizophrenia. When adjusted for smoking status and cumulative dose of clozapine, which were significantly associated with clozapine concentration, SNPs in the *CYP2D6* (rs28371726 and rs202102799), *UGT1A1* (rs4148323 and rs34946978), and *UGT1A4* (rs2011404) genes were significantly associated with clozapine concentration. Further, when adjusted for patients’ follow-up time and BMI, which were significant factors for the total PANSS score, two SNPs each in *ABCB1* (rs7787082 and rs10248420) and *DRD4* (rs2133251840 and rs762502) were significantly associated with the total PANSS score.

Similar to previous findings [16,17], the patients’ smoking status and cumulative dosage of clozapine were significantly associated with clozapine concentrations. Smoking increases the metabolism of clozapine and its metabolite NDMC through the induction of drug-metabolizing enzymes, particularly CYP1A2 and UGT1A4 [30], leading to lower concentrations of clozapine and NDMC in smokers than that in non-smokers. Sex and age have also been reported to influence clozapine concentrations [15,31,]. However, in our study of relatively young patients, age was negatively associated with the clozapine concentration, while sex showed no significant association.

We found that the CYP1A2 and CYP2C19 phenotypes were significantly associated with the metabolic ratio of clozapine at visits 4 and 2, respectively. An *in-vitro* study on clozapine N-demethylation suggested that CYP1A2 plays a major role at low concentrations, while CYP2C19 is of considerable importance at higher concentrations [32]. This finding indicates that individual genetic differences may play a key role in clozapine metabolism, particularly at varying dosage levels or time points during the treatment period. Furthermore, our model indicates that some genetic variants in the *CYP* and *UGT* genes, particularly in *CYP2D6*, *UGT1A1*, and *UGT1A4*, may contribute to the variability in the concentrations of clozapine or its metabolite. However, further large-scale studies are needed to confirm this hypothesis and better understand the clinical implications of these genetic variations.

Our study showed that BMI and some metabolic indicators, including blood levels of glucose, insulin, and HOMA-IR, were negatively associated with the total PANSS score. This aligns with previous findings suggesting a link between weight gain and metabolic changes and treatment response to antipsychotic medications [33,34]. Our study found a positive association between total bilirubin levels and the PANSS score, suggesting a potential link between oxidative stress and psychiatric symptoms, as previously reported [35,36]. Given the insufficient evidence to support the clinical use of total bilirubin levels as a predictive factor for the PANSS, further studies are warranted to clarify the association between bilirubin levels and the severity of psychotic symptoms in schizophrenia.

We identified five SNPs in the *ABCB1*, *DRD4*, and *COMT* genes that were significantly associated with the PANSS score. Two SNPs (rs7787082 and rs10248420), which are located in the introns of *ABCB1* and are in linkage disequilibrium [37], have been suggested as candidate genes for treatment response to clozapine [37]. Genetic polymorphisms in *DRD4*, such as the 48-bp variant number tandem repeat polymorphism or 120-bp tandem repeat polymorphism, may influence clozapine response [38,39]. Given the key role of the COMT enzyme in dopamine degradation [40], the significant association between rs4818 and the total PANSS score in our model suggests that this SNP may serve as a potential genetic marker for treatment response to antipsychotics in patients with schizophrenia, consistent with findings from previous studies [40,41].

This study had some limitations. Both progression of disorder and response to medication may influence the PANSS score, but we were unable to distinguish between their effects. This means that the associations between the PANSS and specific variables observed in our study may not be definitive for some patients, depending on their status of disorder, medications, or both. In addition, owing to the relatively small sample size, some patient characteristics, such as age, were not variable enough to be fully considered for our prediction models. In addition, we included various collected variables in our model, and there is a possibility that some observed results, such as the relationship between total bilirubin and the PANSS, were derived because such diverse variables were considered. To confirm their clinical significance, additional validation in a larger population is necessary. Moreover, there were few genetic variations in some of the SNPs (i.e., rs2011404), resulting in most patients having the same genotype. Therefore, our models need to be validated in larger populations before the findings can be applied in clinical practice. Nevertheless, we could identify factors associated with the clozapine concentration and the total PANSS score in patients receiving clozapine treatment with flexible dosing in actual clinical settings. The predictors of treatment response to clozapine identified in the current study may help in the individualizing of clozapine dosing strategies.

## Conclusions

We found significant clinical and genetic factors impacting clozapine levels and psychiatric symptoms in patients with schizophrenia. Key findings included the influence of smoking and the cumulative dose of clozapine on clozapine levels and the association of specific SNPs in *CYP2D6*, *UGT1A1*, and *UGT1A4* with clozapine levels. Further, total PANSS scores were significantly associated with follow-up time, BMI, and total bilirubin levels. Additionally, some SNPs in the *ABCB1*, *DRD4*, and *COMT* genes were potential predictors of the total PANSS scores. Further investigations in larger populations with diverse characteristics can help confirm our findings for predicting the factors affecting clozapine concentrations and psychiatric symptoms in patients receiving clozapine treatment.

## Supporting information

S1 Table Plasma concentrations of clozapine and N-desmethylclozapine by patient characteristics. (DOCX)

S2 Table Genotypes and phenotypes of CYP1A2 and CYP2C19. (DOCX)

S3 Table Number of patients using concomitant psychotropic agents during each visit interval. (DOCX)

S4 Table Pharmacokinetics-related single nucleotide polymorphisms examined for associations with the clozapine concentration in the linear mixed model. (DOCX)

S5 Table Pharmacodynamics-related single nucleotide polymorphisms examined for association with the Positive and Negative Syndrome Scale score in the linear mixed model. (DOCX)

S6 Table Total Positive and Negative Syndrome Scale score by genotype. (DOCX)

S1 Fig Metabolic ratio of clozapine at (A) visit 2 and (B) visit 4 by CYP1A2 phenotype. *  Indicates statistical significance (p < 0.05) by the Mann–Whitney U test. NM, normal metabolizer; UM, ultrarapid metabolizer.
(TIF)

S2 Fig Metabolic ratio of clozapine at each visit by CYP2C19 phenotype. (A) NM versus IM versus PM at visit 2, (B) NM +  IM versus PM at visit 2, (C) NM versus IM versus PM at visit 4, and (D) NM +  IM versus PM at visit 4. *  Indicates statistical significance (p < 0.05). The Kruskal–Wallis test is used for comparisons between three groups and the Mann–Whitney U test is used for comparisons between two groups. IM, intermediate metabolizer; NM, normal metabolizer; PM, poor metabolizer
(TIF)
