# Supplementary material for: Predictors of clozapine concentration and psychiatric symptoms in patients with schizophrenia
Source: PLoS One. 2025 Mar 6;20(3):e0319037. doi: 10.1371/journal.pone.0319037 (PMC11884701; doi:10.1371/journal.pone.0319037)
Supplement: S1 Table — (DOCX) [file pone.0319037.s001.docx]

**S1 Table. Plasma concentrations of clozapine and N-desmethylclozapine by patient characteristics.**

| **Characteristic** |  | **Sex** | | | **Smoking** | | | **CYP1A2 phenotype** | | |
| --- | --- | --- | --- | --- | --- | --- | --- | --- | --- | --- |
|  | Total | Male | Female | *P*-value^a^ | Smoker | Non-smoker | *P*-value^a^ | UMs | NMs | *P*-value^a^ |
| Visit 2 | *n* = 44^b^ | *n* = 17 | *n* = 27 |  | *n* = 8 | *n* = 36 |  | *n* = 28 | *n* = 16 |  |
| Average daily clozapine dose (mg/day)^c^ | 76.4 ± 36.1 | 78.5 ± 44.9 | 75.1 ± 30.2 | 0.766 | 62.9 ± 46.0 | 79.4 ± 33.6 | 0.181 | 81.5 ± 37.2 | 67.5 ± 33.4 | 0.218 |
| Clozapine concentration (ng/mL) | 243.4 ± 177.4 | 250.7 ± 198.7 | 238.9 ± 166.5 | 0.849 | 94.2 ± 77.1 | 276.6 ± 176.8 | **0.001**^*^ | 255.2 ± 171.4 | 222.9 ± 191.4 | 0.418 |
| NDMC concentration (ng/mL) | 186.3 ± 128.5 | 170.0 ± 136.4 | 196.5 ± 124.7 | 0.327 | 76.6 ± 54.5 | 210.7 ± 127.7 | **0.003**^*^ | 196.3 ± 126.1 | 168.7 ± 134.7 | 0.538 |
| Metabolic ratio^d^ | 1.6 ± 1.1 | 1.9 ± 1.4 | 1.4 ± 0.9 | 0.157 | 1.3 ± 0.6 | 1.6 ± 1.2 | 0.501 | 1.6 ± 1.2 | 1.6 ± 1.0 | 0.570 |
| Visit 3 | *n* = 43^e^ | *n* = 17 | *n* = 26 |  | *n* = 9 | *n* = 34 |  | *n* = 27 | *n* = 16 |  |
| Average daily clozapine dose (mg/day)^c^ | 153.6 ± 120.9 | 182.6 ± 171.8 | 134.6 ± 68.6 | 0.499 | 112.3 ± 58.0 | 164.5 ± 131.1 | 0.126 | 169.8 ± 141.9 | 126.2 ± 68.9 | 0.236 |
| Clozapine concentration (ng/mL) | 302.8 ± 173.0 | 264.3 ± 196.3 | 328.0 ± 154.8 | 0.148 | 178.6 ± 130.2 | 335.7 ± 169.4 | **0.007*** | 302.0 ± 176.2 | 304.1 ± 173.2 | 1.000 |
| NDMC concentration (ng/mL) | 295.1 ± 194.2 | 271.5 ± 185.4 | 310.5 ± 201.8 | 0.649 | 165.2 ± 146.0 | 329.5 ± 192.3 | **0.011*** | 327.3 ± 212.2 | 240.7 ± 149.9 | 0.198 |
| Metabolic ratio^d^ | 1.3 ± 1.0 | 1.0 ± 0.5 | 1.5 ± 1.2 | 0.424 | 1.3 ± 0.9 | 1.3 ± 1.0 | 0.781 | 1.1 ± 0.6 | 1.7 ± 1.4 | 0.165 |
| Visit 4 | *n* = 40^f^ | *n* = 17 | *n* = 23 |  | *n* = 10 | *n* = 30 |  | *n* = 26 | *n* = 14 |  |
| Average daily clozapine dose (mg/day)^c^ | 180.3 ± 95.3 | 200.0 ± 113.8 | 165.8 ± 78.4 | 0.286 | 142.0 ± 47.4 | 193.1 ± 104.1 | 0.142 | 187.3 ± 107.9 | 167.4 ± 67.8 | 0.977 |
| Clozapine concentration (ng/mL) | 404.9 ± 272.5 | 407.6 ± 331.5 | 402.9 ± 227.3 | 0.958 | 290.1 ± 194.2 | 443.2 ± 286.5 | 0.125 | 379.7 ± 274.1 | 451.6 ± 273.3 | 0.433 |
| NDMC concentration (ng/mL) | 353.2 ± 230 | 303.8 ± 214.7 | 389.7 ± 238.7 | 0.248 | 204.4 ± 133.8 | 402.8 ± 235.4 | **0.016**^*^ | 364.4 ± 239.1 | 332.4 ± 219.2 | 0.681 |
| Metabolic ratio^d^ | 1.2 ± 0.7 | 1.4 ± 0.9 | 1.1 ± 0.4 | 0.286 | 1.6 ± 1.0 | 1.1 ± 0.5 | 0.063 | 1.0 ± 0.5 | 1.5 ± 0.8 | **0.006**^*^ |

Data are presented as mean ± standard deviation. ^*^Bold values denote statistical significance (*P* < 0.05).

^a^Mann–Whitney U test or Student’s *t*-test were used, as appropriate.

^b^One patient whose NDMC concentration was less than the limit of quantification was excluded.

^c^The cumulative dose at each visit divided by the number of days from the baseline (visit 1) to the respective visit.

^d^Clozapine/NDMC ratio

^e^Two patients who dropped out were excluded.

^f^One patient whose clozapine and NDMC concentrations were less than the limit of quantification, and four patients who dropped out were excluded.

NDMC, N-desmethylclozapine; NMs, normal metabolizers; UMs, ultrarapid metabolizers
