# Supplementary material for: Predictors of clozapine concentration and psychiatric symptoms in patients with schizophrenia
Source: PLoS One. 2025 Mar 6;20(3):e0319037. doi: 10.1371/journal.pone.0319037 (PMC11884701; doi:10.1371/journal.pone.0319037)
Supplement: S2 Table — (DOCX) [file pone.0319037.s002.docx]

**S2 Table. Genotypes and phenotypes of CYP1A2 and CYP2C19.**

| **SNP** | | | | **Genotype** | **Phenotype** | **Number (%) of patients** |
| --- | --- | --- | --- | --- | --- | --- |
| **CYP1A2** | | | | | | |
| rs2069514 | | rs762551 | |  |  |  |
| GG | | CC | | *1A/*1A | NM | 8 (17.8) |
| AA | | AA | | *1C*1F/*1C*1F | NM | 2 (4.4) |
| GA | | CA | | *1C/*1F or *1A/*1C*1F | NM | 7 (15.6) |
| GG | | AA | | *1F/*1F | UM | 4 (8.9) |
| GA | | AA | | *1F/*1C*1F | UM | 10 (22.2) |
| GG | | CA | | *1A/*1F | UM | 14 (31.1) |
| **CYP2C19** | | | | | | |
| rs12769205 | rs4244285 | | rs3758580 |  |  |  |
| AA | GG | | CC | *1/*1 | NM | 20 (44.4) |
| AG | GA | | CT | *1/*2 | IM | 19 (42.2) |
| GG | AA | | TT | *2/*2 | PM | 6 (13.3) |

IM, intermediate metabolizer; NM, normal metabolizer; PM, poor metabolizer; SNP, single nucleotide polymorphism; UM: ultrarapid metabolizer
