# Supplementary material for: Predictors of clozapine concentration and psychiatric symptoms in patients with schizophrenia
Source: PLoS One. 2025 Mar 6;20(3):e0319037. doi: 10.1371/journal.pone.0319037 (PMC11884701; doi:10.1371/journal.pone.0319037)
Supplement: S3 Table — (DOCX) [file pone.0319037.s003.docx]

**S3 Table. Number of patients using concomitant psychotropic agents during each visit interval.**

|  | **Visit intervals (number of patients, %)** | | | |
| --- | --- | --- | --- | --- |
|  | **Prior to Visit 1** | **Visit 1–2** | **Visit 2–3** | **Visit 3–4** |
| **Antipyschotics** | (n, %) | (n, %) | (n, %) | (n, %) |
| Amisulpride | 12 (26.67%) | 10 (22.22%) | 5 (11.63%) | 6 (14.63%) |
| Aripiprazole | 18 (40.00%) | 17 (37.78%) | 14 (32.56%) | 11 (26.83%) |
| Blonanserin | 3 (6.67%) | 2 (4.44%) | 1 (2.33%) | 0 |
| Olanzapine | 17 (37.78%) | 15 (33.33%) | 12 (27.91%) | 7 (17.07%) |
| Paliperidone | 10 (22.22%) | 9 (20.00%) | 4 (9.30%) | 3 (7.32%) |
| Risperidone | 12 (26.67%) | 9 (20.00%) | 9 (20.93%) | 4 (9.76%) |
| Ziprasidone | 1 (2.22%) | 1 (2.22%) | 1 (2.33%) | 1 (2.44%) |
| Chlorpromazine | 1 (2.22%) | 1 (2.22%) | 1 (2.33%) | 0 |
| Haloperidol | 1 (2.22%) | 3 (6.67%) | 2 (4.65%) | 1 (2.44%) |
| Quetiapine | 15 (33.33%) | 9 (20.00%) | 6 (13.95%) | 5 (12.20%) |
| **Antidepressants** |  |  |  |  |
| Escitalopram | 0 | 8 (17.78%) | 8 (18.60%) | 11 (26.83%) |
| Fluvoxamine | 1 (2.22%) | 1 (2.22%) | 1 (2.33%) | 1 (2.44%) |
| Paroxetine | 0 | 1 (2.22%) | 1 (2.33%) | 1 (2.44%) |
| Sertraline | 1 (2.22%) | 1 (2.22%) | 1 (2.33%) | 3 (7.32%) |
| Trazodone | 0 | 1 (2.22%) | 1 (2.33%) | 1 (2.44%) |
| Vortioxetine | 0 | 1 (2.22%) | 1 (2.33%) | 1 (2.44%) |
| **Anxiolytics** |  |  |  |  |
| Alprazolam | 0 | 6 (13.33%) | 2 (4.65%) | 2 (4.88%) |
| Buspirone | 0 | 1 (2.22%) | 1 (2.33%) | 1 (2.44%) |
| Clonazepam | 0 | 11 (24.44%) | 8 (18.60%) | 8 (19.51%) |
| Diazepam | 0 | 1 (2.22%) | 0 | 0 |
| Lorazepam | 0 | 29 (64.44%) | 26 (60.47%) | 22 (53.66%) |
| **Mood Stabilizers** |  |  |  |  |
| Lithium | 0 | 4 (8.89%) | 4 (9.30%) | 4 (9.76%) |
| Topiramate | 0 | 1 (2.22%) | 1 (2.33%) | 1 (2.44%) |
| Valproate | 0 | 8 (17.78%) | 8 (18.60%) | 7 (17.07%) |
| **Others** |  |  |  |  |
| Benztropine | 0 | 11 (24.44%) | 11 (25.58%) | 14 (34.15%) |
| Chlorpheniramine | 1 (2.22%) | 0 | 0 | 0 |
| Propranolol | 0 | 20 (44.44%) | 20 (46.51%) | 22 (53.66%) |
| Trihexyphenidyl | 0 | 27 (60.00%) | 31 (72.09%) | 29 (70.73%) |

The number of patients who completed visits 1, 2, 3, and 4, were 45, 45, 43, and 41 respectively. The percentages in parentheses were calculated based on the number of people who completed each visit.
