# Supplementary material for: Predictors of clozapine concentration and psychiatric symptoms in patients with schizophrenia
Source: PLoS One. 2025 Mar 6;20(3):e0319037. doi: 10.1371/journal.pone.0319037 (PMC11884701; doi:10.1371/journal.pone.0319037)
Supplement: S4 Table — (DOCX) [file pone.0319037.s004.docx]

**S4 Table. Pharmacokinetics-related single nucleotide polymorphisms examined for associations with the clozapine concentration in the linear mixed model.**

| **Gene** | **SNP ID** | **RSID** | **LD ^a^** | **Chr. Position ^b^** | **Base change ^c^** | **AA change** |
| --- | --- | --- | --- | --- | --- | --- |
| *CYP1A2* | PK_01 | rs2069514 |  | 15:75,038,220 | c.-3860G>A |  |
|  | PK_02 | rs2069521 | a | 15:75,038,967 | c.-3113G>A |  |
|  | PK_03 | rs2069522 | a | 15:75,039,233 | c.-2847T>C |  |
|  | PK_04 | rs762551 |  | 15:75,041,917 | c.-9-154C>A |  |
|  | PK_05 | rs551386466 |  | 15:75,042,490 | c.411G>A | p.Arg137Arg |
|  | PK_06 | rs55918015 |  | 15:75,044,489 | c.1067G>A | p.Arg356Gln |
|  | PK_07 | rs45486893 |  | 15:75,047,191 | c.1313C>T | p.Thr438Ile |
|  | PK_08 | rs2470890 |  | 15:75,047,426 | c.1548C>T | p.Asn516Asn |
| *CYP2D6* | PK_09 | rs1135840 | b | 22:42,522,613 | c.1457C>G | p.Thr486Ser |
|  | PK_10 | rs149157808 |  | 22:42,522,916 | c.1252G>A | p.Glu418Lys |
|  | PK_11 | rs778760893 |  | 22:42,523,484 | c.1129_1138delATGACATCCC | p.Met377fs |
|  | PK_12 | rs150552908 |  | 22:42,523,505 | c.1117G>A | p.Gly373Ser |
|  | PK_13 | rs61745683 |  | 22:42,523,514 | c.1108G>A | p.Val370Ile |
|  | PK_14 | NA |  | 22:42,523,521 | c.1096_1100dupTTTGG | p.Asp368fs |
|  | PK_15 | rs28371726 |  | 22:42,523,539 | c.1083T>C | p.His361His |
|  | PK_16 | rs202102799 |  | 22:42,523,558 | c.1064A>G | p.Tyr355Cys |
|  | PK_17 | rs3915951 |  | 22:42,523,636 | c.986G>T | p.Arg329Leu |
|  | PK_18 | rs28371725 |  | 22:42,523,805 | n.*2451C>T |  |
|  | PK_19 | rs16947 | c | 22:42,523,943 | c.886T>C | p.Cys296Arg |
|  | PK_20 | rs79331140 |  | 22:42,524,369 | n.*3015G>C |  |
|  | PK_21 | rs79738337 |  | 22:42,524,490 | n.*3136G>A |  |
|  | PK_22 | rs58440431 | d | 22:42,524,696 | n.*3342T>C |  |
|  | PK_23 | rs111564371 | e | 22:42,524,708 | n.*3354T>C |  |
|  | PK_24 | rs112568578 | e | 22:42,524,713 | n.*3359C>G |  |
|  | PK_25 | rs113889384 | e | 22:42,524,743 | n.*3389G>A |  |
|  | PK_26 | rs28371713 | e | 22:42,524,795 | c.657T>C | p.Phe219Phe |
|  | PK_27 | rs111606937 |  | 22:42,524,924 | c.528T>C | p.Gly176Gly |
|  | PK_28 | rs5030865 |  | 22:42,525,035 | c.505G>A | p.Gly169Arg |
|  | PK_29 | rs1058164 | b | 22:42,525,132 | c.408C>G | p.Val136Val |
|  | PK_30 | rs1081003 | d | 22:42,525,756 | c.336C>T | p.Phe112Phe |
|  | PK_31 | rs28371702 | b | 22:42,525,952 | n.*4598C>A |  |
|  | PK_32 | NA |  | 22:42,526,038 | n.*4684G>C |  |
|  | PK_33 | rs28371701 | c | 22:42,526,049 | n.*4695C>G |  |
|  | PK_34 | rs1931831820 |  | 22:42,526,061 | n.*4707T>G |  |
|  | PK_35 | NA |  | 22:42,526,063 | n.*4709G>T |  |
|  | PK_36 | rs28371699 | b | 22:42,526,484 | c.180+130T>G |  |
|  | PK_37 | rs1081000 |  | 22:42,526,549 | c.180+65G>A |  |
|  | PK_38 | rs28695233 | c | 22:42,526,561 | c.180+53C>A |  |
|  | PK_39 | rs75276289 | c | 22:42,526,562 | c.180+52C>G |  |
|  | PK_40 | rs76312385 | c | 22:42,526,567 | c.180+47C>T |  |
|  | PK_41 | rs74644586 | c | 22:42,526,571 | c.180+43G>C |  |
|  | PK_42 | rs1080996 | c | 22:42,526,573 | c.180+41A>C |  |
|  | PK_43 | rs1080995 | c | 22:42,526,580 | c.180+34C>G |  |
|  | PK_44 | rs1065852 | d | 22:42,526,694 | c.100C>T | p.Pro34Ser |
| *CYP3A5* | PK_45 | rs28365085 |  | 7:99,245,974 | c.1463T>C | p.Ile488Thr |
|  | PK_46 | rs6977165 |  | 7:99,269,397 | c.423A>G | p.Ter141Trpext*? |
|  | PK_47 | rs776746 |  | 7:99,270,539 | n.689-1G>A |  |
| *CYP3A4* | PK_48 | rs772477178 |  | 7:99,361,484 | c.1020C>A | p.Pro340Pro |
|  | PK_49 | rs28371759 |  | 7:99,361,626 | c.878T>C | p.Leu293Pro |
|  | PK_50 | rs4646437 |  | 7:99,365,083 | c.671-202C>T |  |
|  | PK_51 | rs12721627 |  | 7:99,366,093 | c.554C>G | p.Thr185Ser |
|  | PK_52 | rs55951658 |  | 7:99,367,825 | c.352A>G | p.Ile118Val |
| *CYP2C19* | PK_53 | rs17885098 |  | 10:96,522,561 | c.99T>C | p.Pro33Pro |
|  | PK_54 | rs12769205 | f | 10:96,535,124 | c.332-23A>G |  |
|  | PK_55 | rs4986893 |  | 10:96,540,410 | c.636G>A | p.Trp212* |
|  | PK_56 | rs4244285 | f | 10:96,541,616 | c.681G>A | p.Pro227Pro |
|  | PK_57 | rs3758580 | f | 10:96,602,622 | c.990C>T | p.Val330Val |
|  | PK_58 | rs3758581 |  | 10:96,602,623 | c.991G>A | p.Val331Ile |
|  | PK_59 | rs17886522 |  | 10:96,609,775 | c.1251A>C | p.Gly417Gly |
| *UGT1A4* | PK_60 | rs2011425 | g | 2:234,627,608 | c.142T>G | p.Leu48Val |
|  | PK_61 | rs757477070 | h | 2:234,627,640 | c.175delG | p.Val59fs |
|  | PK_62 | rs201323245 |  | 2:234,627,758 | c.292C>T | p.Gln98* |
|  | PK_63 | rs539093785 | h | 2:234,627,791 | c.325A>G | p.Arg109Gly |
|  | PK_64 | rs12468274 | g | 2:234,627,914 | c.448T>C | p.Leu150Leu |
|  | PK_65 | rs2011404 |  | 2:234,627,937 | c.471T>C | p.Cys157Cys |
|  | PK_66 | rs3732217 | g | 2:234,628,270 | c.804G>A | p.Pro268Pro |
| *UGT1A3* | PK_67 | rs28898617 |  | 2:234,637,789 | c.17A>G | p.Gln6Arg |
|  | PK_68 | rs3821242 | i | 2:234,637,803 | c.31T>C | p.Trp11Arg |
|  | PK_69 | rs6706232 | i | 2:234,637,853 | c.81G>A | p.Glu27Glu |
|  | PK_70 | rs45625338 |  | 2:234,637,905 | c.133C>T | p.Arg45Trp |
|  | PK_71 | rs6431625 | j | 2:234,637,912 | c.140T>C | p.Val47Ala |
|  | PK_72 | rs7574296 | i | 2:234,638,249 | c.477A>G | p.Ala159Ala |
| *UGT1A1* | PK_73 | rs887829 | j | 2:234,668,570 | n.-4164G>A |  |
|  | PK_74 | rs4148323 |  | 2:234,669,144 | c.211G>A | p.Gly71Arg |
|  | PK_75 | rs35350960 |  | 2:234,669,619 | c.686C>A | p.Pro229Gln |
|  | PK_76 | rs34946978 |  | 2:234,676,872 | c.1082C>T | p.Pro361Leu |
| *UGT1A8* | PK_77 | rs199539868 |  | 2:234,681,094 | c.1482C>T | p.Ala494Ala |
| *UGT2B10* | PK_78 | rs376619171 |  | 4:69,682,219 | c.482A>C | p.Glu161Ala |
|  | PK_79 | rs1976666 |  | 4:69,683,875 | c.847C>G | p.Pro283Ala |
|  | PK_80 | rs61301802 |  | 4:69,692,163 | c.1035C>G | p.Ala345Ala |
|  | PK_81 | rs117559713 |  | 4:69,696,568 | c.1558A>G | p.Arg520Gly |

^a^ SNP pairs with r^2^ values > 0.8, denoted by a−j.

^b^All coordinate positions are in accordance with the UCSC genomic build GRCh37/hg19.

^c^ Nucleotide location numbers are assigned according to *CYP1A2* (NM_000761.3), *CYP2D6* (NM_000106.5), *CYP3A5* (NM_000777.3, NM_001190484.1, NR_033807.1), *CYP3A4* (NM_017460.5), *CYP2C19* (NM_000769.1), *UGT1A4* (NM_007120.2), *UGT1A3* (NM_019093.2), *UGT1A1* (NM_000463.2), *UGT1A8* (NM_019076.4), and *UGT2B10* (NM_001075.4) mRNA sequences.

AA, amino acid; Chr, chromosome; LD, linkage disequilibrium; NA, not applicable
