# Supplementary material for: Predictors of clozapine concentration and psychiatric symptoms in patients with schizophrenia
Source: PLoS One. 2025 Mar 6;20(3):e0319037. doi: 10.1371/journal.pone.0319037 (PMC11884701; doi:10.1371/journal.pone.0319037)
Supplement: S5 Table — (DOCX) [file pone.0319037.s005.docx]

**S5 Table. Pharmacodynamics-related single nucleotide polymorphisms examined for association with the Positive and Negative Syndrome Scale score in the linear mixed model.**

| **Gene** | **SNP ID** | **RSID** | **LD ^a^** | **Chr. Position ^b^** | **Base change ^c^** | **AA change** |
| --- | --- | --- | --- | --- | --- | --- |
| *HRH1* | PD_01 | rs17034063 |  | 3:11,206,322 | c.-36+27237C>T |  |
|  | PD_02 | rs1552498 |  | 3:11,230,464 | c.-36+51379G>A |  |
|  | PD_03 | rs13064530 |  | 3:11,265,914 | c.-34810G>A |  |
| *DRD3* | PD_04 | rs167771 |  | 3:113,876,275 | c.383+2327C>T |  |
|  | PD_05 | rs138354054 |  | 3:113,890,728 | c.112G>A | p.Ala38Thr |
|  | PD_06 | rs3732783 |  | 3:113,890,789 | c.51A>G | p.Ala17Ala |
|  | PD_07 | rs6280 |  | 3:113,890,815 | c.25G>A | p.Gly9Ser |
| *TNIK* | PD_08 | rs2088885 |  | 3:170,971,291 | c.124-25281G>T |  |
| *SLC6A3* | PD_09 | rs974803762 |  | 5:1,409,877 | c.1357G>A | p.Val453Ile |
|  | PD_10 | rs6347 |  | 5:1,411,412 | c.1215A>G | p.Ser405Ser |
|  | PD_11 | rs200132776 |  | 5:1,432,658 | c.574G>A | p.Ala192Thr |
|  | PD_12 | rs6351 |  | 5:1,443,151 | c.162C>T | p.Pro54Pro |
|  | PD_13 | rs2975226 |  | 5:1,445,616 | c.-2304T>A |  |
| *DRD1* | PD_14 | rs1799914 |  | 5:174,869,905 | c.198G>A | p.Leu66Leu |
|  | PD_15 | rs4532 |  | 5:174,870,150 | c.-48G>A |  |
| *DTNBP1* | PD_16 | rs777114347 |  | 6:15,524,726 | c.842C>T | p.Pro281Leu |
|  | PD_17 | rs742105 |  | 6:15,573,074 | c.511+20216G>A |  |
| *ABCB1* | PD_18 | rs1045642 |  | 7:87,138,645 | c.3435T>C | p.Ile1145Ile |
|  | PD_19 | rs763091787 |  | 7:87,138,695 | c.3385G>A | p.Glu1129Lys |
|  | PD_20 | rs144369247 |  | 7:87,148,696 | c.2873G>A | p.Arg958Gln |
|  | PD_21 | rs7787082 | a | 7:87,157,051 | c.2685+3559C>T |  |
|  | PD_22 | rs2032582 |  | 7:87,160,618 | c.2677T>G | p.Ser893Ala |
|  | PD_23 | rs2032582 |  | 7:87,160,618 | c.2677T>A | p.Ser893Thr |
|  | PD_24 | rs10248420 | a | 7:87,164,986 | c.2481+788T>C |  |
|  | PD_25 | rs1128503 |  | 7:87,179,601 | c.1236T>C | p.Gly412Gly |
|  | PD_26 | rs189559454 |  | 7:87,199,501 | c.325G>A | p.Glu109Lys |
|  | PD_27 | rs199607036 |  | 7:87,199,522 | c.304G>C | p.Gly102Arg |
|  | PD_28 | rs3747802 |  | 7:87,342,586 | c.-440T>C |  |
| *DRD4* | PD_29 | rs572586776 |  | 11:637,362 | c.76_87delGCATCTGCGGGG | p.Ala26_Gly29del |
|  | PD_30 | rs778114005 |  | 11:639,956 | c.707G>C | p.Arg236Pro |
|  | PD_31 | rs2133251840 |  | 11:639,959 | c.710_711delGA | p.Arg237fs |
|  | PD_32 | rs1003315598 | b | 11:639,959 | c.710G>C | p.Arg237Pro |
|  | PD_33 | rs2133251845 | b | 11:639,960 | c.711A>C | p.Arg237Arg |
|  | PD_34 | rs2133251861 |  | 11:639,964 | c.715_716delAG | p.Ser239fs |
|  | PD_35 | rs2133251864 |  | 11:639,964 | c.715_719delAGCGG | p.Ser239fs |
|  | PD_36 | rs1258037201 | c | 11:639,964 | c.715A>C | p.Ser239Arg |
|  | PD_37 | rs2133251867 | c | 11:639,965 | c.716G>C | p.Ser239Thr |
|  | PD_38 | rs537247984 |  | 11:639,967 | c.718G>C | p.Gly240Arg |
|  | PD_39 | rs771071064 |  | 11:639,968 | c.719G>C | p.Gly240Ala |
|  | PD_40 | rs1474769762 |  | 11:639,972 | c.723T>C | p.Pro241Pro |
|  | PD_41 | rs770385372 |  | 11:639,974 | c.725G>C | p.Gly242Ala |
|  | PD_42 | NA |  | 11:640,004 | c.807_902delTCCCCGGGGTCCCTGCGGCCCCGACTGTGCGCCCGCCGCGCCCAGCCTCCCCCAGGACCCCTGCGGCCCCGACTGTGCGCCCCCCGCGCCCGGCCT | p.Pro270_Leu301del |
|  | PD_43 | NA |  | 11:640,023 | c.801_802insAGCCTCCCCCAGGACCCCTGTGGCCCCGACTGTGCGCCCCCCGCGCCC | p.Pro267_Gly268insSerLeuProGlnAspProCysGlyProAspCysAlaProProAlaPro |
|  | PD_44 | rs767239460 |  | 11:640,061 | c.812G>A | p.Arg271Gln |
|  | PD_45 | NA |  | 11:640,066 | c.841_888delGCCGCGCCCAGCCTCCCCCAGGACCCCTGCGGCCCCGACTGTGCGCCC | p.Ala281_Pro296del |
|  | PD_46 | rs34662058 |  | 11:640,099 | c.850A>G | p.Ser284Gly |
|  | PD_47 | rs762502 |  | 11:640,119 | c.870C>T | p.Cys290Cys |
| *ANKK1* | PD_48 | rs17115439 |  | 11:113,264,272 | c.255T>C | p.Ser85Ser |
|  | PD_49 | rs58224139^d^ |  | 11:113,264,467 | c.450C>T | p.Asn150Asn |
|  | PD_50 | rs4938013 |  | 11:113,264,470 | c.453A>C | p.Ile151Ile |
|  | PD_51 | rs7118900 | d | 11:113,266,821 | c.715G>A | p.Ala239Thr |
|  | PD_52 | rs11604671 | e | 11:113,268,059 | c.952G>A | p.Gly318Arg |
|  | PD_53 | rs4938016 |  | 11:113,270,015 | c.1324G>C | p.Gly442Arg |
|  | PD_54 | rs2734849 | e | 11:113,270,160 | c.1469A>G | p.His490Arg |
|  | PD_55 | rs147474296 |  | 11:113,270,369 | c.1678G>A | p.Gly560Ser |
|  | PD_56 | rs2734848 |  | 11:113,270,374 | c.1683C>T | p.Tyr561Tyr |
|  | PD_57 | NA |  | 11:113,270,710 | c.2019G>C | p.Leu673Leu |
|  | PD_58 | rs1800497 | d | 11:113,270,828 | c.2137G>A | p.Glu713Lys |
| *DRD2* | PD_59 | rs6277 |  | 11:113,283,459 | c.957C>T | p.Pro319Pro |
|  | PD_60 | rs6275 |  | 11:113,283,477 | c.939T>C | p.His313His |
|  | PD_61 | rs1801028 |  | 11:113,283,484 | c.932C>G | p.Ser311Cys |
|  | PD_62 | rs201422791 |  | 11:113,283,486 | c.930G>A | p.Pro310Pro |
| *HTR2A* | PD_63 | rs6313 |  | 13:47,469,940 | c.102C>T | p.Ser34Ser |
| *AKT1* | PD_64 | rs3001371 |  | 14:105,242,831 | c.287+165G>A |  |
| *SLC6A2* | PD_65 | rs5569 |  | 16:55,731,835 | c.1287G>A | p.Thr429Thr |
| *COMT* | PD_66 | rs4633 |  | 22:19,950,235 | c.186C>T | p.His62His |
|  | PD_67 | rs6267 |  | 22:19,950,263 | c.214G>T | p.Ala72Ser |
|  | PD_68 | rs4818 |  | 22:19,951,207 | c.408C>G | p.Leu136Leu |
|  | PD_69 | rs4680 |  | 22:19,951,271 | c.472G>A | p.Val158Met |
|  | PD_70 | rs769224 |  | 22:19,951,804 | c.597G>A | p.Pro199Pro |
|  | PD_71 | rs1233424008 |  | 22:19,956,218 | c.775A>G | p.Lys259Glu |
|  | PD_72 | rs1327994695 |  | 22:19,956,219 | c.776A>C | p.Lys259Thr |
| *HTR2C* | PD_73 | rs3813928 | f | X:113,818,282 | c.-143064G>A |  |
|  | PD_74 | rs3813929 | f | X:113,818,520 | c.-142826C>T |  |
|  | PD_75 | rs1556486977 |  | X:114,141,371 | c.770C>G | p.Pro257Arg |

^a^ SNP pairs with r^2^ values > 0.8, denoted by a−j.

^b^All coordinate positions are in accordance with the UCSC genomic build GRCh37/hg19.

^c^ Nucleotide location numbers are assigned according to *HRH1* (NM_001098213.1), *DRD3* (NM_000796.3), *TNIK* (NM_015028.2), *SLC6A3* (NM_001044.4), *DRD1* (NM_000794.3), *DTNBP1* (NM_183040.2), *DTNBP1* (NM_032122.4), *ABCB1* (NM_000927.4), *DRD4* (NM_000797.3), *DRD2* (NM_000795.3), *HTR2A* (NM_000621.4), *AKT1* (NM_001014431.1), *SLC6A2* (NM_001172504.1), *COMT* (NM_000754.3), and *HTR2C* (NM_000868.2) mRNA sequences.

^d^ rs58224139 was excluded in the analysis because all patients are wild-type.

AA, amino acid; Chr, chromosome; LD, linkage disequilibrium
