# Supplementary material for: Predictors of clozapine concentration and psychiatric symptoms in patients with schizophrenia
Source: PLoS One. 2025 Mar 6;20(3):e0319037. doi: 10.1371/journal.pone.0319037 (PMC11884701; doi:10.1371/journal.pone.0319037)
Supplement: S6 Table — (DOCX) [file pone.0319037.s006.docx]

**S6 Table. Total Positive and Negative Syndrome Scale score by genotype.**

| **Gene** | **SNP** | **Genotype (N)^a^** | **Baseline (visit 1)** | **Week 8 (visit 3)** | **Change from baseline PANSS score at visit 3 (%)** | **Week 18 (visit 4)** | **Change from baseline PANSS score at visit 4 (%)** |
| --- | --- | --- | --- | --- | --- | --- | --- |
| *ABCB1* | rs7787082^b^ | AA (16) | 83.44 ± 14.06 | 77.75 ± 12.35 | -6.56 ± 6.07 | 71.56 ± 12.80 | -14.06 ± 8.07 |
|  |  | AB+BB (23) | 80.78 ± 17.74 | 73.43 ± 13.05 | -7.44 ± 14.51 | 67.22 ± 9.62 | -14.40 ± 16.00 |
|  |  | *p*-value | 0.466^c^ | 0.306 | 0.549^c^ | 0.234 | 0.931 |
|  | rs10248420^b^ | AA (16) | 83.44 ± 14.06 | 77.75 ± 12.35 | -6.56 ± 6.07 | 71.56 ± 12.80 | -14.06 ± 8.07 |
|  |  | AB+BB (23) | 80.78 ± 17.74 | 73.43 ± 13.05 | -7.44 ± 14.51 | 67.22 ± 9.62 | -14.40 ± 16.00 |
|  |  | *p*-value | 0.466^c^ | 0.306 | 0.549^c^ | 0.234 | 0.931 |
| *DRD4* | rs2133251840 | AA (30) | 79.30 ± 15.24 | 72.43 ± 12.31 | -7.92 ± 9.20 | 66.17 ± 9.74 | -15.25 ± 11.65 |
|  |  | AB (9) | 90.44 ± 17.18 | 84.44 ± 10.10 | -4.29 ± 18.19 | 78.44 ± 10.47 | -10.96 ± 17.84 |
|  |  | *p*-value | 0.121^c^ | **0.008*^c^** | 0.582^c^ | **0.003*^c^** | 0.395^c^ |
|  | rs2133251864 | AA (24) | 85.92 ± 17.10 | 78.04 ± 13.91 | -7.98 ± 13.72 | 70.50 ± 11.75 | -16.36 ± 14.56 |
|  |  | AB (15) | 75.40 ± 12.56 | 70.67 ± 9.49 | -5.63 ± 7.62 | 66.60 ± 9.84 | -10.91 ± 10.19 |
|  |  | *p*-value | 0.103^c^ | 0.079 | 0.273^c^ | 0.291 | 0.214 |
|  | rs762502 | AA (3) | 89.33 ± 25.81 | 84.67 ± 21.36 | -4.62 ± 3.57 | 71.00 ± 3.61 | -16.08 ± 23.19 |
|  |  | AB+BB (36) | 81.25 ± 15.53 | 74.42 ± 11.96 | -7.28 ± 12.13 | 68.83 ± 11.51 | -14.11 ± 12.56 |
|  |  | *p*-value | 0.635^c^ | 0.429^c^ | 0.445^c^ | 0.544^c^ | 0.693^c^ |
| *COMT* | rs4818 | AA (18) | 77.78 ± 15.30 | 72.89 ± 13.21 | -5.12 ± 14.38 | 65.56 ± 8.16 | -13.67 ± 15.33 |
|  |  | AB+BB (21) | 85.38 ± 16.46 | 77.19 ± 12.38 | -8.76 ± 8.81 | 71.95 ± 12.54 | -14.77 ± 11.40 |
|  |  | *p*-value | 0.159^c^ | 0.301 | 0.693^c^ | 0.072 | 0.799 |

Data are reported as mean ± SD for continuous variables and n (%) for categorical variables.

*P*-values are computed by the t-test or Mann–Whitney U test, as appropriate. *****Bold values denote statistical significance (*p* < 0.05).

AA, AB, and BB indicate wild-type, heterozygous, and homozygous mutant group, respectively.

^a^ Patients who completed PANSS assessment from baseline to visit 4 (N=39) were included.

^b^ Linkage disequilibrium with r^2^ > 0.8.

^c^ Mann–Whitney U test

PANSS, Positive and Negative Syndrome Scale; SNP, single nucleotide polymorphism
