# Supplementary figures and images for: Predictors of clozapine concentration and psychiatric symptoms in patients with schizophrenia
Source: PLoS One. 2025 Mar 6;20(3):e0319037. doi: 10.1371/journal.pone.0319037 (PMC11884701; doi:10.1371/journal.pone.0319037)

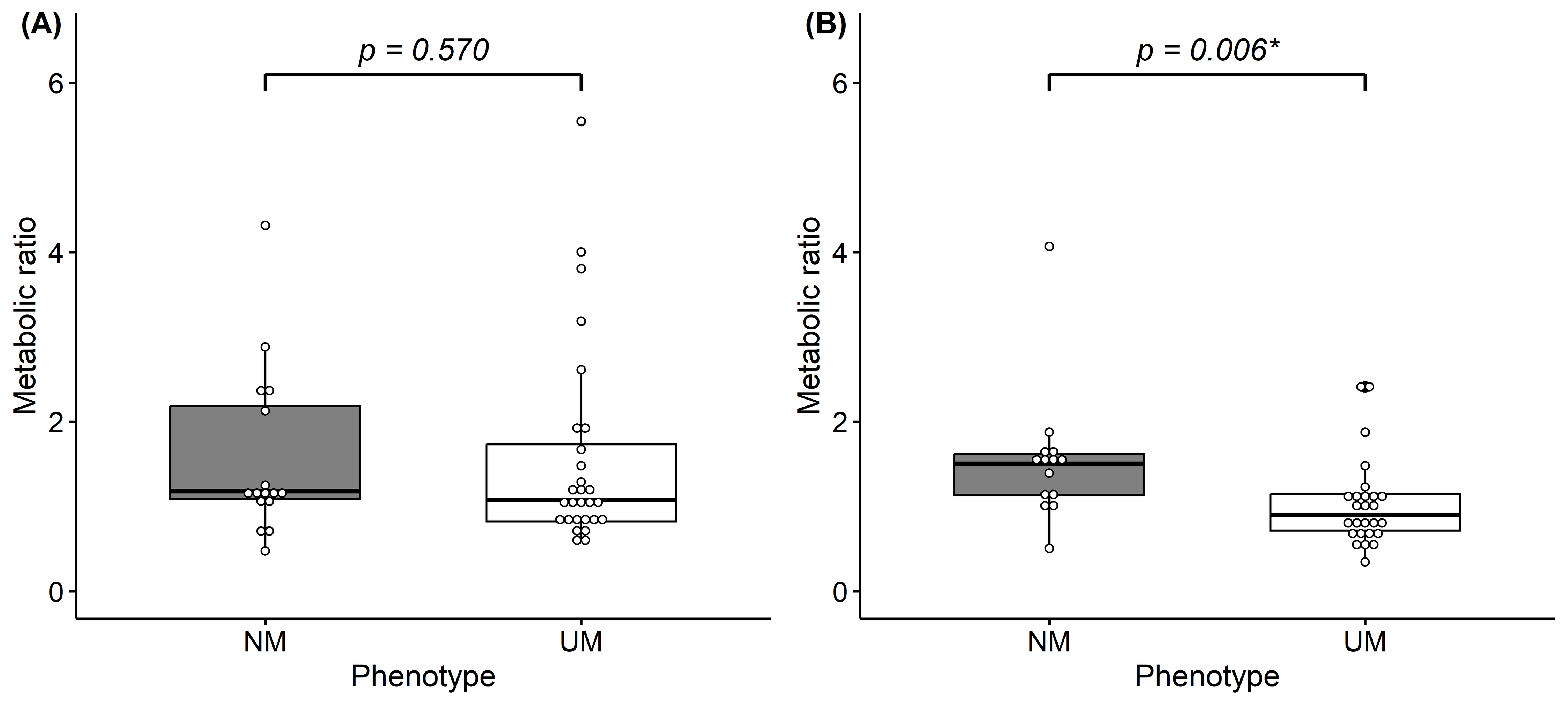

Supplement: S1 Fig — (A) visit 2 and (B) visit 4 by CYP1A2 phenotype. * Indicates statistical significance (p < 0.05) by the Mann–Whitney U test. NM, normal metabolizer; UM, ultrarapid metabolizer. (TIF) [file pone.0319037.s007.tif]

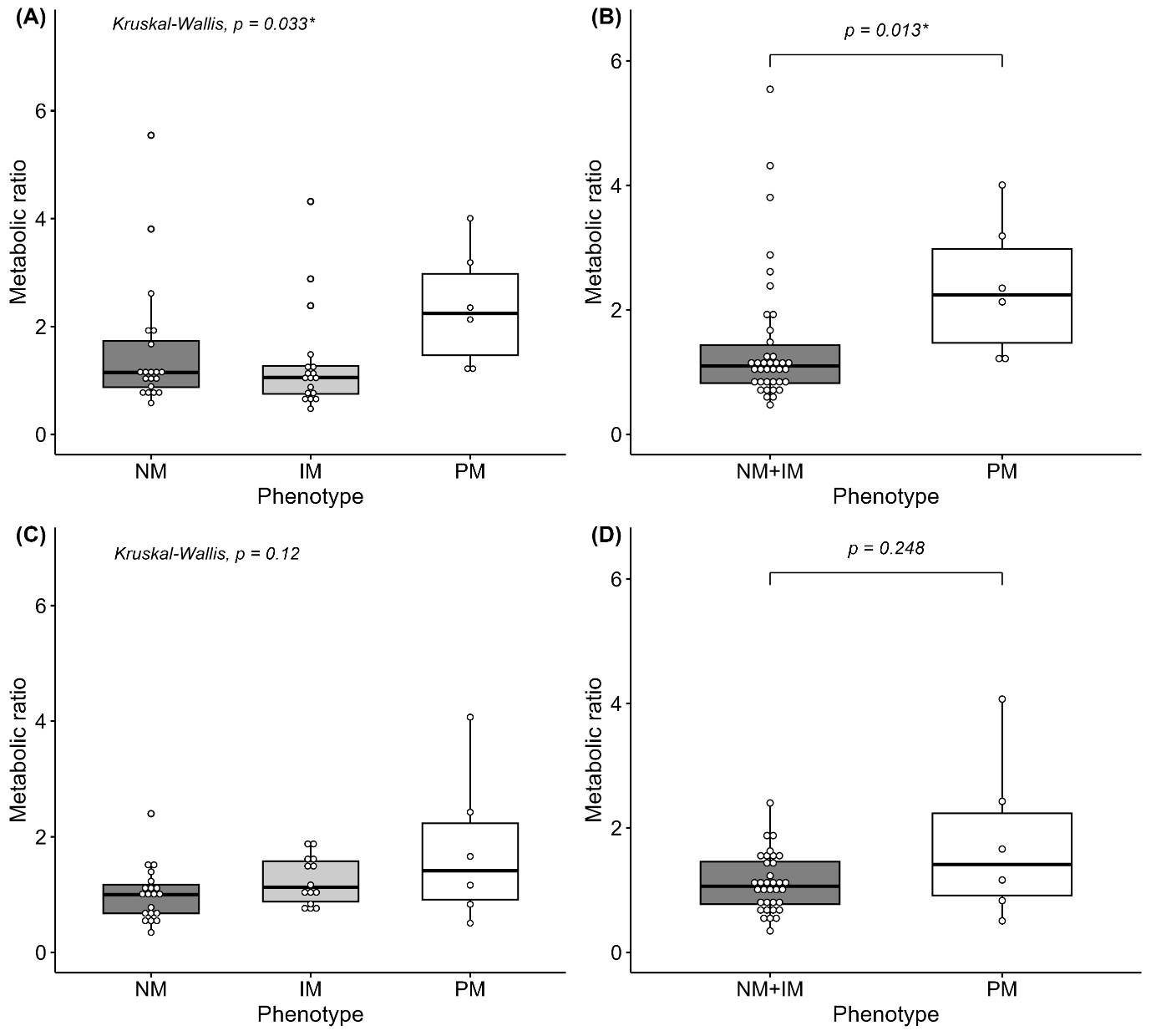

Supplement: S2 Fig — (A) NM versus IM versus PM at visit 2, (B) NM + IM versus PM at visit 2, (C) NM versus IM versus PM at visit 4, and (D) NM + IM versus PM at visit 4. * Indicates statistical significance (p < 0.05). The Kruskal–Wallis test is used for comparisons between three groups and the Mann–Whitney U test is used for comparisons between two groups. IM, intermediate metabolizer; NM, normal metabolizer; PM, poor metabolizer (TIF) [file pone.0319037.s008.tif]
